# Ventilation dynamics using a portable device coupled to the six-minute walk test in people with long-COVID syndrome: a preliminary study

**DOI:** 10.1186/s13104-023-06374-3

**Published:** 2023-06-08

**Authors:** Jéssica Gabriela Messias Oliveira, Renan Pereira Campos, Beatriz Luiza Pinheiro Alves Azevedo, Samantha Gomes de Alegria, Patrícia Frascari Litrento, Thiago Thomaz Mafort, Agnaldo José Lopes

**Affiliations:** 1 Department of Pulmonology, Universidade do Estado do Rio de Janeiro (UERJ), Policlínica Piquet Carneiro, Avenida Mal. Rondon, 381, São Francisco Xavier, Rio de Janeiro, 20950-003 Brazil; 2 Post-Graduation Programme in Rehabilitation Sciences, Centro Universitário Augusto Motta (UNISUAM), Rua Dona Isabel, 94, Bonsucesso, Rio de Janeiro, 21032-060 Brazil; 3 Post-Graduation Programme in Medical Sciences, School of Medical Sciences, Universidade do Estado do Rio de Janeiro (UERJ), Boulevard 28 de Setembro, 77, Vila Isabel, Rio de Janeiro, 20551-030 Brazil

**Keywords:** Long-COVID syndrome, Six-minute walk test, Pulmonary function testing, Cardiopulmonary exercise test, Small airway disease

## Abstract

**Objective:**

To investigated the dynamic ventilatory responses and their influence on functional exercise capacity in patients with long-COVID-19 syndrome (LCS).

**Results:**

Sixteen LCS patients were subjected to resting lung function (spirometry and respiratory oscillometry-RO) and cardiopulmonary performance to exercise (Spiropalm®-equipped six-minute walk test-6MWT and cardiopulmonary exercise test-CPX). At rest, spirometry showed a normal, restrictive and obstructive pattern in 87.5%, 6.25% and 6.25% of participants, respectively. At rest, RO showed increased resonance frequency, increased integrated low-frequency reactance and increased difference between resistance at 4–20 Hz (R4-R20) in 43.7%, 50%, and 31.2% of participants, respectively. The median of six-minute walking distance (DTC6) was 434 (386–478) m, which corresponds to a value of 83% (78–97%) of predicted. Dynamic hyperinflation (DH) and reduced breathing reserve (BR) were detected in 62.5% and 12.5% of participants, respectively. At CPX, the median peak oxygen uptake (VO_2peak_) was 19 (14–37) ml/kg/min. There was a significant correlation of 6MWD with both R4-R20 (r_s_=-0.499, *P* = 0.039) and VO_2peak_ (r_s_=0.628, *P* = 0.009). Our results indicate that DH and low BR are contributors to poor exercise performance, which is associated with peripheral airway disease. These are promising results considering that they were achieved with simple, portable ventilatory and metabolic systems.

## Introduction

There is much information regarding the acute process caused by new coronavirus. The marked inflammatory response and the coagulopathy state caused by COVID-19 may promote lung damage. In addition, acute new coronavirus infection is not limited to the lungs and has multisystem effects, with evidence of important cardiovascular disorders [1]. However, there is scarce information concerning the effect of the disease on functional exercise capacity in long-COVID syndrome (LCS), especially regarding the dynamic changes in ventilation, as the lungs are the most affected organs [2].

The ventilatory limitation during exercise is usually assessed by the breathing reserve (BR), which indicates how close the minute ventilation (VE) approaches the maximum ventilation during a given activity [3]. Another measure used to evaluate ventilatory mechanics is dynamic hyperinflation (DH), which refers to exercise-induced air trapping [4]. As the respiratory rate (RR) increases during exercise, expiratory time decreases, limiting the ability to completely empty the alveoli. When a new breath is initiated before the alveoli have been completely emptied, the end-expiratory lung volume increases, consequently restricting the inspiratory capacity (IC) during effort [4].

Besides the deconditioning, persistent low-grade inflammation after acute new coronavirus infection may contribute to systemic problems, which supports the need for further evaluation of cardiopulmonary fitness, especially in the most severely affected patients, including evaluation of functional exercise capacity [1]. In fact, it is considered that traditional measures of pulmonary function, such as forced expiratory volume in one second (FEV_1_), may not reflect the seriousness of lung involvement and functional impairment in LCS [2, 5]. Consequently, more specific measures of pulmonary function, such as DH or BR, may increase understanding of the mechanisms of exercise intolerance. Although patients with LCS have evidence of peripheral airway disease (PAD) [6], no previous study has investigated DH in this population using the six-minute walk test (6MWT). Our goal was to investigate the dynamic ventilatory responses and their impact on the functional capacity to exercise in people with LCS.

## Main text

### Methods

A cross-sectional analysis was conducted between March and October 2022 with 16 COVID-19 survivors (out of 19 eligible patients) aged ≥ 18 years old attending the Policlínica Piquet Carneiro, Universidade do Estado do Rio de Janeiro, Rio de Janeiro, Brazil. Patients who suffered from COVID-19 pneumonia with persistence of respiratory symptoms after 3 months [7] and who had not been hospitalized at the time of acute COVID-19 were included. Patients with a history of smoking or chronic lung disease, and those who could not execute the protocol tests were excluded. The Research Ethics Committee of the Hospital Universitário Pedro Ernesto approved the project under the number CAAE-30135320.0.0000.5259, and all participants signed the consent form.

Respiratory oscillometry (RO) was carried out employing an appropriate device (Quark i2m, Cosmed, Rome, Italy). At the time of RO assessment, the subjects were asked to remain seated, with manual support on the cheeks, and nostrils occluded by a clip, and to breathe normally for 40 s. We analyse the following indexes: respiratory system resistance (Rrs) at 4 Hz (R4) and 20 Hz (R20); mean resistance between 4 and 20 Hz (Rm); difference between resistance at 4–20 Hz (R4-R20); resonance frequency (Fres); and integrated low-frequency reactance (AX). The next values were deemed abnormal: R4 and/or R20 ≥ 150% of predicted; Fres > 12 Hz; AX > 3.60 cm H_2_O/L/s; and R4-R20 > 20%, which has also been utilized for the diagnosis of PAD [8, 9]. After five minutes of performing the RO, the Vitatrace VT device (Codax Ltda, Rio de Janeiro, Brazil) was used to perform spirometry, employing national reference values [10].

The 6MWT was performed according to previous guidelines [11], with a silicone face mask of the portable device (Spiropalm 6MWT, Cosmed, Rome, Italy) attached to the patient. Before and at the end of the test, the inspiratory capacity (IC) was measured. A decrease of ≥ 100 ml in IC (∆IC) during exercise was defined as DH [4]. Besides DH, other dynamic ventilatory responses were measured, including minute ventilation (VE) and breathing reserve (BR). BR indicates how close VE approaches maximum ventilation during a given activity and was calculated as the difference between maximal voluntary ventilation (MVV) and VE_peak_ ([MVV-VE_peak_]/MVV) [12]; BR < 30% was considered ventilatory limitation on exertion [4]. MVV was automatically determined by the device as 40 multiplied by the patient’s measured FEV_1_ [12]. The device also measured the heart rate (HR) and the oxygen saturation (SO_2_), and a decrease of ≥ 4% in SO_2_ was considered desaturation. The predicted values for the six-minute walking distance (6MWD) were obtained from the reference equation [13].

Lastly, the subjects underwent cardiopulmonary exercise test (CPX) limited by symptoms, according to previous recommendations [14]. Briefly, the test was conducted with the use of a breath-by-breath system on a cycle ergometer connected to a FitMate™ (Cosmed, Rome, Italy) calibrated according to the manufacturer’s specifications. The FitMate™ utilizes new sampling technology using a small sample representative of the exhaled volume in a miniaturized dynamic mixing chamber. The FitMate™ does not have a CO_2_ analyser and has software that increases the respiratory exchange ratio between 0.8 and 1.2 based on the increase in HR [15]. Exercise was stopped when the subjects developed marked dyspnoea or muscle fatigue and were exhausted.

A nonparametric method was employed as the variables did not present a normal (Gaussian) distribution, according to the rejection of the hypothesis of normality using the Shapiro-Wilk test. The inferential analysis consisted of the Spearman correlation coefficient for the association between lung function at rest and cardiopulmonary exercise performance. The significance level adopted was the 5% level. Statistical evaluation was processed using SPSS statistical software version 26.

## Results

Nineteen LCS patients were evaluated to be included in the study; three, however, were excluded because of walking difficulties. All participants had moderate COVID according to World Health Organization definitions [16]; none of them used corticosteroids and/or bronchodilators at the time of acute COVID or subsequently underwent pulmonary rehabilitation. At the time of acute COVID, 10 and 6 patients had a percentage of lung parenchymal involvement ˂25% and between 25 and 50% on computed tomography scans, respectively. The median age and time since COVID-19 diagnosis were 57 (50–59) years and 98 (93–106) days, respectively. Fourteen (87.5%) participants were women, with 12 (75%) of them having a BMI ≥ 30 kg/m^2^ [median body mass index of 32 (30–36) kg/m^2^]. Regarding lung function at rest, spirometry showed a normal, restrictive and obstructive pattern in 14 (87.5%), 1 (6.25%) and 1 (6.25%) individual, respectively. In RO, there were Fres > 12 Hz and AX > 3.60 cm H_2_O/L/s in 7 (43.7%) and 8 (50%) cases, respectively. The R4 and/or R20 values were ≥ 150% in 7 (43.7%) cases, and an R4-R20 value > 20% was detected in 5 (31.2%) cases. Considering the abnormalities in the resistive and reactive indexes, 8 (50%) participants had abnormal RO. Table 1 presents the clinical data and results of pulmonary function at rest.

**Table 1:** Clinical data and Lung function at rest

Variables	Values
Clinical data	
Hypertension	6 (37.5%)
Diabetes	4 (25%)
Heart disease	2 (12.5%)
Spirometry	
FVC (% predicted)	93 (88–103)
FEV_1_ (% predicted)	96 (88–102)
FEV_1_/FVC (%)	84 (76–89)
FEF_25 − 75%_ (% predicted)	114 (74–126)
Respiratory oscillometry	
Fres (Hz)	11.8 (9–20)
Rm (cm H_2_O/L/s)	5.6 (3.2–6.7)
R4 (cm H_2_O/L/s)	6.3 (5.3–8)
R4 (% predicted)	141 (112–194)
R20 (cm H_2_O/L/s)	5.4 (4.6–6.6)
R20 (% predicted)	136 (108–186)
R4-R20 (cm H_2_O/L/s)	0.7 (0.2–2.2)
AX (cm H_2_O/L)	3.6 (1.5–4.8)

*FVC* forced vital capacity, *FEV*_*1*_ forced expiratory volume in one second, *FEF*_*25 − 75%*_ forced expiratory flow during the middle half of the FVC, *Fres* resonance frequency, Rm = mean resistance between 4–20 Hz, *R4* resistance at 4 Hz, *R20* resistance at 20 Hz, *R4-R20* difference between resistance at 4–20 Hz, *AX* integrated low-frequency reactance

Results are expressed as median (interquartile ranges) or number (%)

Regarding cardiopulmonary exercise performance (Table 2), the median 6MWD during exercise Spiropalm®-equipped 6MWT was 83% (78–97%) predicted, with 5 (31.2%) patients experiencing a 6MWD < 80% predicted. DH was noted in 10 (62.5%) subjects. Two (12.5%) participants had ventilatory limitation (BR < 30%) and desaturation during the 6MWT. In the FitMate™ testing, the median of the peak oxygen uptake (VO_2peak_) was 19 (14–37) ml/kg/min.

**Table 2:** Cardiopulmonary performance to exercise

Variables	Values
Spiropalm®-equipped 6MWT	
6MWD (m)	434 (386–478)
6MWD (% predicted)	83 (78–97)
Basal SO_2_ (%)	96 (93–98)
End of test SO_2_ (%)	94 (92–96)
Basal HR (pulse/min)	87 (79–92)
End of test HR (pulse/min)	116 (108–119)
Resting VE (L/min)	14 (12–16)
VE_peak_ (L/min)	30 (26–36)
BR (%)	62 (52–69)
Basal IC (L)	2.3 (2–2.7)
End of test IC (L)	2.1 (1.7–2.5)
∆IC (L)	-0.2 (-0.5–-0.1)
FitMate™ testing	
VO_2peak_ (ml/kg/min)	19 (14–37)
VE_peak_ (L/min)	47 (38–69)
End of test RR (breaths/min)	37 (34–46)
End of test HR (pulse/min)	138 (123–161)

*6MWT* six-minute walk test, *6MWD* six‐minute walking distance, *SO*_*2*_ oxygen saturation, *HR* heart rate, *VE* minute ventilation, *BR* breathing reserve, *IC* inspiratory capacity, *VO*_*2peak*_ peak oxygen uptake, *RR* respiratory rate

Results are expressed as median (interquartile ranges)

**Fig. 1:**
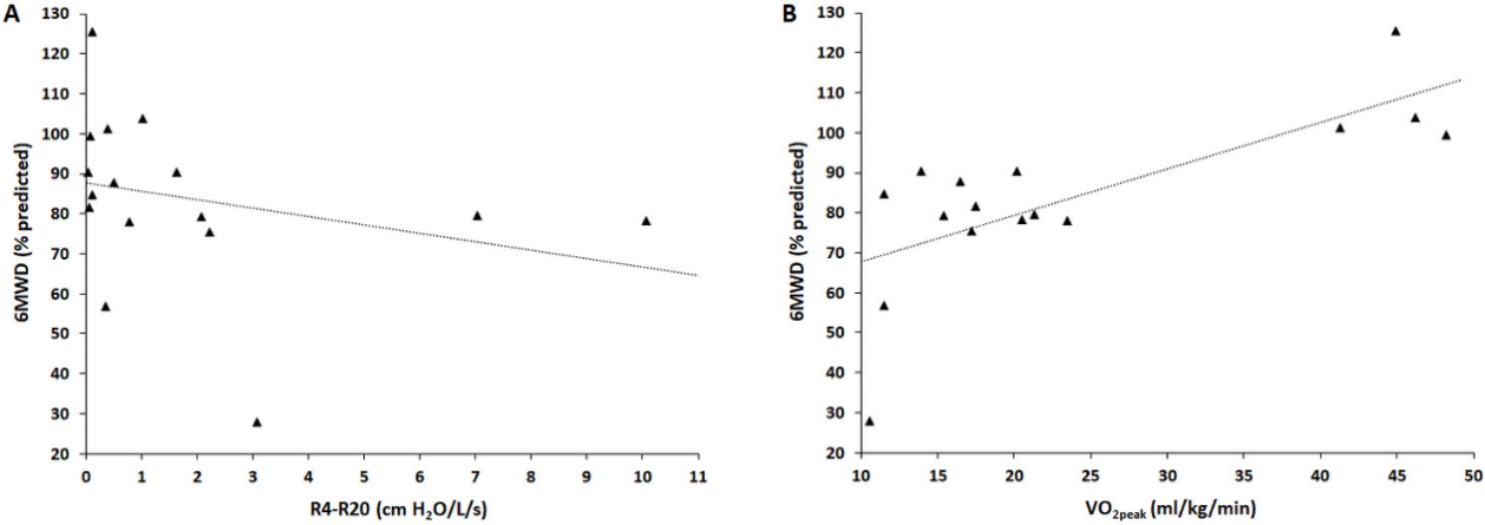
Relationship of six-minute walking distance (6MWD) with the difference between resistance at 4–20 Hz (R4-R20, *r*_*s*_=-0.499, *P* = 0.039) **(A)** and the peak oxygen uptake (VO_2peak_, *r*_*s*_=0.628, *P* = 0.009) **(B)**

The associations between resting lung function and cardiopulmonary exercise performance are demonstrated in Table 3. The 6MWD was significantly correlated with both R4-R20 and VO_2peak_ (Fig. 1).

**Table 3:** Spearman correlation coefficient between lung function at rest and cardiopulmonary performance to exercise

Variables		R4-R20	6MWD	BR	∆IC	VO_2peak_
FVC	*r*_*s*_	-0.055	0.075	-0.150	-0.047	0.191
	*P*	0.84	0.78	0.58	0.85	0.48
R4-R20	*r*_*s*_	-	**-0.499**	-0.075	0.134	-0.185
	*P*	-	**0.039**	0.78	0.62	0.49
6MWD	*r*_*s*_	-	-	0.046	-0.110	**0.628**
	*P*	-	-	0.87	0.68	**0.009**
BR	*r*_*s*_	-	-	-	0.080	0.003
	*P*	-	-	-	0.77	0.99
∆IC	*r*_*s*_	-	-	-	-	0.289
	*P*	-	-	-	-	0.28

*FVC* forced vital capacity, *R4-R20* difference between resistance at 4–20 Hz, *AX* integrated low-frequency reactance, *6MWD* six-minute walking distance, *BR* breathing reserve, *IC* inspiratory capacity, *VO*_*2peak*_ peak oxygen uptake

Bold type indicates significant correlations

## Discussion

A simple, efficient, low-cost method that allows assessing cardiopulmonary fitness during submaximal exercise is of great interest due to COVID-19’s impact on the lungs. Measuring dynamic ventilation during the 6MWT in individuals with LCS, the major findings of this study were that more than 60% of them showed DH, although only a small portion showed ventilatory limitation and significant desaturation. Furthermore, there was an association of 6MWD with both oxygen consumption and the presence of PAD. According to our knowledge, our study is the first to find a relationship between DH and PAD in LCS patients who did not require hospitalization at the time of acute infection.

In the analysed sample, almost one-third of the subjects had a predicted 6MWD < 80%. The median 6MWD was 434 m, which is close to the values reported by Townsend et al. [1] and González et al. [17] in individuals with LCS but greater than 350 m; the latter value has been associated with all-cause mortality in subjects with preexisting lung disease [18]. We noted that just over 10% of the participants had ventilatory limitation using BR < 30%, which is a parameter traditionally used to assess ventilatory limitation since it is a simple technique that does not require additional analysis. Although BR is commonly used to assess ventilatory limitation, it does not offer data about the possible mechanisms of ventilatory restriction or the respiratory strategy of patients during exercise [4]. Thus, the investigation of DH helps to better explain the pathophysiology involved in ventilatory limitation and intolerance to effort. Using ∆CI to define DH, we found that more than 60% of participants had DH. It is worth noting that DH is a strong determinant of exercise tolerance because it increases the mechanical load on the inspiratory muscles and impairs the ability of tidal volume to increase adequately with exercise [4].

Regarding sensitivity, OR has proved to be an effective test for the accurate assessment of PAD in LCS individuals, as new coronavirus can lead to airway calibre reduction, bronchiolitis, bronchiolocentric interstitial pneumonia and peribronchial remodelling [6, 19]. Like other studies [6, 20], we noticed that half of our subjects had RO changes, including abnormalities compatible with PAD. Curiously, we noticed an association between 6MWD and R4-R20, which is a sensitive index for the diagnosis of PAD. Thus, one can speculate that the inhomogeneity in the distribution of ventilation as measured by Rrs may be an important contributor to the lower tolerance to exercise in LCS patients. It is worth noting that these individuals may have other important limitations that prevent their level of exercise from increasing, such as general fatigue, muscle weakness, psychological changes, or damage to the pulmonary circulation, which may even explain the desaturation observed during exercise in part of these patients [21].

In our sample, 75% of patients were obese, which is not surprising given that obesity is a risk factor for COVID-19 [22]. Since obesity itself has a negative impact on the RO indexes (respiratory compliance and resistance), many of the abnormalities observed in our study can be explained by the excess of adipose tissue around the upper airways. Conjointly, the impairment of peripheral resistance may modify the performance in the 6MWT. In fact, Perossi et al. [23] found associations between the RO indexes and 6MWD, suggesting that PAD may be related to worsening functional capacity in women with severe obesity. Since both obesity and LPS are conditions that express several pro-inflammatory markers, we think that inflammation is a possible link that should be better studied in obese individuals with LPS.

Technological advances led to the development of portable indirect calorimeters, which are lightweight, battery-operated, and capable of measuring VO_2peak_. Consequently, there has been a trend towards the use of portable breath-by-breath analysers in an attempt to link respiratory variables to metabolic events in the muscle [24]. Using a portable breath-by-breath analyser, we demonstrated a strong correlation between VO_2peak_ and 6MWD. VO_2peak_ is a reliable measure of cardiorespiratory fitness and, therefore, a marker of the maximum capacity of the oxidative system to provide energy during exercise [25]. Using the same device to assess dynamic ventilation during the 6MWT in individuals with fibrosis of the lungs (which is the final route of pulmonary repair in a significant number of LCS patients), De Martino et al. [26] observed that ventilation increased significantly because of the contribution of tidal volume and RR; this finding was associated with a decrease in BR at the end of the test. In comparison with CPX, these authors showed that BR at the end of the 6MWT was also inversely related to peak BR derived from CPX, suggesting BR as fundamental in the assessment of exercise limitation.

In summary, our initial results propose that DH and, to a lesser extent, low BR are contributors to the poor performance of LCS patients during the 6MWT. Furthermore, the worse the PAD and the lower the oxygen consumption, the lower the 6MWD in this patient population. These are promising results, considering that they were attained using simple, inexpensive and portable ventilatory and metabolic measurement systems that are easily applicable in real-world environments.

### Limitations

Our study has some limitations. First, the sample size is small and, therefore, the results obtained should be evaluated with caution. Second, this study was cross-sectional; therefore, we suggest an ongoing evaluation of LCS patients who have persistent health problems. In addition, the participants did not undergo the 6MWT and CPX before COVID-19, and thus, changes from baseline are difficult to assess. Large samples are still needed to determine whether these tools are sensitive enough to identify changes over time.

## Data Availability

All the data supporting the results are provided in the manuscript.

## Abbreviations

6MWD: Six-minute walking distance
6MWT: Six‐minute walk test
AX: Integrated low frequency reactance
BR: Breathing reserve
CPX: Cardiopulmonary exercise test
DH: Dynamic hyperinflation
FEV_1_: Forced expiratory volume in one second
Fres: Resonance frequency
HR: Heart rate
IC: Inspiratory capacity
LCS: Long-COVID syndrome
MVV: Maximal voluntary ventilation
PAD: Peripheral airway disease
R4-R20: Difference between resistance at 4–20 Hz
Rm: Mean resistance between 4 and 20 Hz
RO: Respiratory oscillometry
Rrs: Respiratory system resistance
SO_2_: Oxygen saturation
VE: Minute ventilation
